# Analysis of Information-Based Nonparametric Variable Selection Criteria

**DOI:** 10.3390/e22090974

**Published:** 2020-08-31

**Authors:** Małgorzata Łazęcka, Jan Mielniczuk

**Affiliations:** 1 Institute of Computer Science, Polish Academy of Sciences, Jana Kazimierza 5, 01-248 Warsaw, Poland; m.lazecka@ipipan.waw.pl; 2 Faculty of Mathematics and Information Science, Warsaw University of Technology, Koszykowa 75, 00-662 Warsaw, Poland

**Keywords:** conditional mutual information, CMI, information measures, nonparametric variable selection criteria, gaussian mixture, conditional infomax feature extraction, CIFE, joint mutual information criterion, JMI, generative tree model, Markov blanket

## Abstract

We consider a nonparametric Generative Tree Model and discuss a problem of selecting active predictors for the response in such scenario. We investigated two popular information-based selection criteria: Conditional Infomax Feature Extraction (CIFE) and Joint Mutual information (JMI), which are both derived as approximations of Conditional Mutual Information (CMI) criterion. We show that both criteria CIFE and JMI may exhibit different behavior from CMI, resulting in different orders in which predictors are chosen in variable selection process. Explicit formulae for CMI and its two approximations in the generative tree model are obtained. As a byproduct, we establish expressions for an entropy of a multivariate gaussian mixture and its mutual information with mixing distribution.

## 1. Introduction

In the paper, we consider theoretical properties of Conditional Mutual Information (CMI) and its approximations in a certain dependence model called Generative Tree Model (GTM). CMI and its modifications are used in many problems of machine learning including feature selection, variable importance ranking, causal discovery, and structure learning of dependence networks (see, e.g., Reference [1,2]). They are the cornerstone of nonparametric methods to solve such problems meaning that no parametric assumptions on dependence structure are imposed. However, formal properties of these criteria remain largely unknown. This is mainly due to two problems: firstly, theoretical values of CMI and related quantities are hard to calculate explicitly, especially when the conditioning set has a large dimension. Moreover, there are only a few established facts about behavior of their sample counterparts. Such a situation, however, has important consequences. In particular, a relevant question whether certain information based criteria, such as Conditional Infomax Feature Extraction (CIFE) and Joint Mutual Information (JMI), obtained as approximations of CMI, e.g., by truncation of its Möbius expansion are approximations in analytic sense (i.e., whether the difference of both quantities is negligible) remains unanswered. In the paper, we try to fill this gap. The considered GTM is a model for which marginal distributions of predictors are mixtures of gaussians. Exact values of CMI, as well as of those of CIFE and JMI, are calculated for this model, which makes studying their behavior when parameters of the model and number of predictors change feasible. In particular, it is shown that CIFE and JMI exhibit different behavior than CMI and also they may significantly differ between themselves. In particular, we show, that depending on the value of model parameters, each of considered criteria JMI and CIFE can incorporate inactive variables before active ones into a set of chosen predictors. This, of course, does not mean that important performance criteria, such as False Detection Rate (FDR), cannot be controlled for CIFE and JMI but should serve as a cautionary note that their similarity to CMI, despite their derivation, is not necessarily ensured. As a byproduct, we establish expressions for an entropy of a multivariate gaussian mixture and its mutual information with mixing distribution, which are of independent interest.

We stress that our approach is intrinsically nonparametric and focuses on using nonparametric measures of conditional dependence for feature selection. By studying their theoretical behavior for this task we also learn an average behavior of their empirical counterparts for large sample sizes.

Generative Tree Model appears, e.g., in Reference [3], a non-parametric tree structured model is also considered, e.g., in Reference [4,5]. Together with autoregressive model, it is one of the two most common types of generative models. Besides its easily explainable dependence structure, distributions of predictors in the considered model are mixed gaussians, and this facilitates calculation of explicit form of information-based selection criteria.

The paper is structured as follows. Section 2 contains information-theoretic preliminaries, some necessary facts on information based feature-selection and derivation of CIFE and JMI criteria as approximations of CMI. Section 3 contains derivation of entropy and mutual information for gaussian mixtures. In Section 4, behavior of CMI, CIFE, and JMI is studied in GTM. Section 5 concludes.

## 2. Preliminaries

We denote by p(x), $x\in R^{d}$ a probability density function corresponding to continuous variable *X* on $R^{d}$. Joint density of *X* and variable *Y* will be denoted by p(x,y). In the following, *Y* will denote discrete random response to be predicted using multivariate vector *X*.

Below, we discuss some information-theoretic preliminaries, which leads, at the end of Section 2.1, to Möbius decomposition of mutual information. This is used in Section 2.2 to construct CIFE approximation of CMI. In addition, properties of Mutual Information discussed in Section 2.1 are used in Section 2.2 to justify JMI criterion.

### 2.1. Information-Theoretic Measures of Dependence

The (differential) entropy for continuous random variable *X* is defined as

(1) $$H\left(X\right)=-\int_{R^{d}}p\left(x\right)\log p\left(x\right)\;dx$$

and quantifies the uncertainty of observing random values of *X*. Note that the definition above is valid regardless the dimensionality *d* of the range of *X*. For discrete *X*, we replace the integral in (1) by the sum and density p(x) by probability mass function. In the following, we will frequently consider subvectors of $X=(X_{1},\ldots ,X_{p})$, which is a vector of all potential predictors of discrete response *Y*. The conditional entropy of *X* given discrete *Y* is written as

(2) $$H\left(X|Y\right)=\sum_{y\in Y}p\left(y\right)H\left(X|Y=y\right).$$

When *Z* is continuous, the conditional entropy H(X|Z) is defined as $E_{Z}H\left(X|Z=z\right)$, i.e.,

(3) $$H\left(X|Z\right)=-\int p\left(z\right)\int \frac{p(x,z)}{p(z)}\log\left(\frac{p(x,z)}{p(z)}\right)\;dxdz=-\int p\left(x,z\right)\log\left(\frac{p(x,z)}{p(z)}\right)\;dxdz,$$

where p(x,z) and p(z) denote joint density of (X,Z) and density of *Z*, respectively. The mutual information (MI) between *X* and *Y* is

(4) I(X,Y)=H(X)−H(X|Y)=H(X)−H(Y|X).

This can be interpreted as the amount of uncertainty in *X* (*Y*) which is removed when *Y* (respectively, *X*) is known, which is consistent with the intuitive meaning of mutual information as the amount of information that one variable provides about another. It determines how similar the joint distribution is to the product of marginal distributions when Kullback-Leibler divergence is used as similarity measure (cf. Reference [6], Equation (8.49)). Thus, I(X,Y) may be viewed as nonparametric measure of dependence. Note that, as I(X,Y) is symmetric, it only shows the strength of dependence but not its direction. In contrast to correlation coefficient MI is able to discover non-linear relationships as it equals zero if and only if *X* and *Y* are independent. It is easily seen that I(X,Y)=H(X)+H(Y)−H(X,Y). A natural extension of MI is conditional mutual information (CMI) defined as

(5) $$I\left(X,Y|Z\right)=H\left(X|Z\right)-H\left(X|Y,Z\right)=\int p\left(z\right)\int p\left(x,y|z\right)\log\frac{p(x,y|z)}{p(x|z)p(y|z)}\;dxdydz,$$

which measures the conditional dependence between *X* and *Y* given *Z*. When *Z* is a discrete random variable, the first integral is replaced by a sum. Note that the conditional mutual information is mutual information of *X* and *Y* given Z=z averaged over values *z* of *Z*, and it equals zero if and only if *X* and *Y* are conditionally independent given *Z*. Important property of MI is a chain rule which connects $I(\left(X_{1},X_{2}\right),Y)$ with $I(X_{1},Y)$:

(6) $$I\left(\left(X_{1},X_{2}\right),Y\right)=I\left(X_{1},Y\right)+I\left(X_{2},Y|X_{1}\right).$$

For more properties of the basic measures described above, we refer to Reference [6,7]. We define now interaction information II ([8]), which is a useful tool for decomposing mutual information between multivariate random variable $X_{S}$ and *Y* (see Formula (13) below). The 3-way interaction information is defined as

(7) $$II\left(X_{1},X_{2},Y\right)=I\left(\left(X_{1},X_{2}\right),Y\right)-I\left(X_{1},Y\right)-I\left(X_{2},Y\right).$$

This is frequently interpreted as the part of $I(\left(X_{1},X_{2}\right),Y)$, which remains after subtraction of individual informations between *Y* and $X_{1}$ and *Y* and $X_{2}$. The definition indicates in particular that $II(X_{1},X_{2},Y)$ is symmetric. Note that it follows from (6) that

(8) $$II\left(X_{1},X_{2},Y\right)=I\left(X_{1},Y|X_{2}\right)-I\left(X_{1},Y\right)=I\left(X_{2},Y|X_{1}\right)-I\left(X_{2},Y\right),$$

which is consistent with the intuitive meaning of existence of interaction as a situation in which the effect of one variable on the class variable *Y* depends on the value of another variable. By expanding all mutual informations on RHS of (7), we obtain

(9) $$II\left(X_{1},X_{2},Y\right)=-H\left(X_{1}\right)-H\left(X_{2}\right)-H\left(Y\right)+H\left(X_{1},Y\right)+H\left(X_{2},Y\right)+H\left(X_{1},X_{2}\right)-H\left(X_{1},X_{2},Y\right).$$

The 3-way II can be extended to the general case of *p* variables. The *p*-way interaction information [9,10] is

(10) $$II\left(X_{1},\ldots ,X_{p}\right)=-\sum_{T\subseteq \{1,\ldots ,p\}}{\left(-1\right)}^{p-|T|}H\left(X_{T}\right).$$

For p=2, (10) reduces to mutual information, whereas, for p=3, it reduces to (9).

We consider two useful properties of introduced measures. We first start with 3-way information interaction, and we note that it inherits chain-rule property from MI, namely

(11) $$II\left(X_{1},\left(X_{2},X_{3}\right),Y\right)=II\left(X_{1},X_{3},Y\right)+II\left(X_{1},X_{2},Y|X_{3}\right),$$

where $I(X_{1},X_{2},Y|X_{3})$ is defined analogously to (7) by replacing mutual informations on RHS by conditional mutual informations given $X_{3}$. This is easily proved by writing, in the view of (6):

(12) $$\begin{array}{ccc} & & II\left(X_{1},\left(X_{2},X_{3}\right),Y\right)=I\left(X_{1},\left(X_{2},X_{3}\right)|Y\right)-I\left(X_{1},\left(X_{2},X_{3}\right)\right)=\\ & & I\left(X_{1},X_{3}|Y\right)+I\left(X_{1},X_{2}|Y,X_{3}\right)-\left[I\left(X_{1},X_{3}\right)+I\left(X_{1},X_{2}|X_{3}\right)\right] \end{array}$$

and using (8) in the above equalities. Namely, joining the first and the third expression together (and the second and the fourth, as well), we obtain that RHS equals $II\left(X_{1},X_{3},Y\right)+II\left(X_{1},X_{2},Y|X_{3}\right)$.

We also state Möbius representation of mutual information which plays an important role in the following development. For S⊆{1,2,…,p}, let $X_{S}$ be a random vector coordinates of which have indices in *S*. Möbius representation [10,11,12] states that $I(X_{S},Y)$ can be recovered from interaction informations

(13) $$I\left(X_{S},Y\right)=\sum_{k=1}^{\left|S\right|}\sum_{\{t_{1},\ldots ,t_{k}\}\subseteq S}II\left(X_{t_{1}},\ldots ,X_{t_{k}},Y\right),$$

where |S| denotes number of elements of set *S*.

### 2.2. Information-Based Feature Selection

We consider discrete class variable *Y* and *p* features $X_{1},\ldots ,X_{p}$. We do not impose any assumptions on dependence between *Y* and $X_{1},\ldots ,X_{p}$, i.e., we view its distributional structure in a nonparametric way. Let $X_{S}$ denote a subset of features, indexed by set S⊆{1,…,p}. As $I(X_{S},Y)$ does not decrease when *S* is replaced by its superset $S^{\prime }⊇S$, the problem of finding ${arg max}_{S}I\left(X_{S},Y\right)$ has a trivial solution full={1,2,…,p}. Thus, one usually tries to optimize mutual information between $X_{S}$ and *Y* under some constraints on the size |S| of *S*. The most intuitive approach is an analogue of *k*-best subset selection in regression which tries to identify a feature subset of a fixed size 1≤k≤p that maximizes the joint mutual information with a class variable *Y*. However, this is infeasible for large *k* because the search space grows exponentially with the number of features. As a result, various greedy algorithms have been developed including forward selection, backward elimination and genetic algorithms. They are based on observation that

(14) $$\underset{j\in S^{c}}{arg max}\left[I\left(X_{S\cup \{j\}},Y\right)-I\left(X_{S},Y\right)\right]=\underset{j\in S^{c}}{arg max}I\left(X_{j},Y|X_{S}\right),$$

where $S^{c}=\left\{1,\ldots ,p\right\}\backslash S$ is a complement of *S*. The equality in (14) follows from (6). In each step, the most promising candidate is added. In the case of ties in (14), the variable satisfying it with the smallest index is chosen.

### 2.3. Approximations of CMI: CIFE and JMI Criteria

Observe that it follows from (13)

(15) $$I\left(X_{S\cup \{j\}},Y\right)-I\left(X_{S},Y\right)=I\left(X_{j},Y|X_{S}\right)=\sum_{k=0}^{\left|S\right|}\sum_{\{t_{1},\ldots ,t_{k}\}\subseteq S}II\left(X_{t_{1}},\ldots ,X_{t_{k}},X_{j},Y\right).$$

Direct application of the above formula to find the maximizer in (14) is infeasible as estimation of a specific information interaction of order *k* requires $O(C^{k})$ observations. The above formula allows us, however, to obtain various natural approximations of CMI. The first order approximation does not take interactions between features into account and that is why the second order approximation obtained by taking first two terms in (15) is usually considered. The corresponding score for candidate feature $X_{j}$ is

(16) $$CIFE\left(X_{j},Y|X_{S}\right)=I\left(X_{j},Y\right)+\sum_{i\in S}II\left(X_{i},X_{j},Y\right)=I\left(X_{j},Y\right)+\sum_{i\in S}\left[I\left(X_{i},X_{j}|Y\right)-I\left(X_{i},X_{j}\right)\right].$$

The acronym CIFE stand for Conditional Infomax Feature Extraction, and the measure has been introduced in Reference [13]. Observe that if interactions of order 3 and higher between predictors are 0, i.e., $II(X_{t_{1}},\ldots ,X_{t_{k}},X_{j},Y)=0$ for k≥2 and then CIFE coincides with CMI. In Reference [2], it is shown that CMI also coincides with CIFE if certain dependence assumptions on vector (X,Y) are satisfied. In view of the discussion above, CIFE can be viewed as a natural approximation to CMI.

Observe that, in (16), we take into account not only relevance of the candidate feature, but also the possible interactions between the already selected features and the candidate feature. The empirical evaluation indicates that (16) is among the most successful MI-based methods; see Reference [2] for an extensive comparison of several MI-based feature selection approaches. We mention in this context, Reference [14], in which stopping rules for CIFE-based methods are considered.

Some additional assumptions lead to other score functions. We show now reasoning leading to Joint Mutual Information Criterion JMI (cf. Reference [12], on which the derivation below is based). Namely, if we define $S=\{j_{1},\ldots ,j_{\left|S\right|}\}$, we have for i∈S

$$I\left(X_{j},X_{S}\right)=I\left(X_{j},X_{i}\right)+I\left(X_{j},X_{S\backslash \{i\}}|X_{i}\right).$$

Summing these equalities over all i∈S and dividing by |S|, we obtain

$$I\left(X_{j},X_{S}\right)=\frac{1}{\left|S\right|}\sum_{i\in S}I\left(X_{j},X_{i}\right)+\frac{1}{\left|S\right|}\sum_{i\in S}I\left(X_{j},X_{S\backslash \{i\}}|X_{i}\right)$$

and analogously

$$I\left(X_{j},X_{S}|Y\right)=\frac{1}{\left|S\right|}\sum_{i\in S}I\left(X_{j},X_{i}|Y\right)+\frac{1}{\left|S\right|}\sum_{i\in S}I\left(X_{j},X_{S\backslash \{i\}}|X_{i},Y\right).$$

Subtracting the two last equations and using (8), we obtain

(17) $$I\left(X_{j},Y|X_{S}\right)=I\left(X_{j},Y\right)+\frac{1}{\left|S\right|}\sum_{i\in S}II\left(X_{j},X_{i},Y\right)+\frac{1}{\left|S\right|}\sum_{i\in S}II\left(X_{j},X_{S\backslash \left\{i\right\}},Y|X_{i}\right).$$

Moreover, it follows from (8) that when $X_{j}$ is independent of $X_{S\backslash \{i\}}$ given $X_{i}$ and these quantities are independent given $X_{i}$ and *Y* the last sum is 0 and we obtain equality

(18) $$JMI\left(X_{j},Y|X_{S}\right)=I\left(X_{j},Y\right)+\frac{1}{\left|S\right|}\sum_{i\in S}II\left(X_{j},X_{i},Y\right)=I\left(X_{j},Y\right)+\frac{1}{\left|S\right|}\sum_{i\in S}\left[I\left(X_{j},X_{i}|Y\right)-I\left(X_{j},X_{i}\right)\right].$$

This is Joint Mutual Information Criterion (JMI) introduced in Reference [15]. Note that (18) together with (8) imply another useful representation

(19) $$JMI\left(X_{j},Y|X_{S}\right)=I\left(X_{j},Y\right)+\frac{1}{\left|S\right|}\sum_{i\in S}\left[I\left(X_{j},Y|X_{i}\right)-I\left(X_{j},Y\right)\right]=\frac{1}{\left|S\right|}\sum_{i\in S}I\left(X_{j},Y|X_{i}\right).$$

JMI can be viewed as an approximation of CMI when independence assumptions on which the above derivation was based are satisfied only approximately. Observe that $JMI(X_{j},Y|X_{S})$ differs from $CIFE(X_{j},Y|X_{S})$ in that the influence of the sum of interaction informations $II(X_{j},X_{i},Y)$ is down weighted by factor ${\left|S\right|}^{-1}$ instead of 1. This is sometimes interpreted as coping with ‘redundancy over-scaled’ problem (cf. Reference [2]). When the terms $I(X_{j},X_{i}|Y)$ are omitted from the sum above then minimal redundancy maximal relevance (mRMR) criterion is obtained [16]. We note that approximations of CMI, such as CIFE or JMI, can be used in place of CMI in (14). As the derivation in both cases is quite intuitive, it is natural to ask how the approximations compare when used for selection. This is the primary aim of the present paper. Theoretical behavior of such methods will be investigated in the following sections. Note that we do not consider empirical counterparts of the above selection rules and investigate how they would behave provided their values have been known exactly.

## 3. Auxiliary Results: Information Measures for Gaussian Mixtures

In the following section, we will prove some results on information-theoretic properties of gaussian mixtures which are necessary to analyze the behavior of CMI, CIFE, and JMI in Generative Tree Model defined below.

In the next section, we will consider a gaussian Generative Tree Model, in which the main components have marginal distributions being mixtures of normal distributions. Namely, if *Y* has Bernoulli distribution $Y∼\mathrm{Bern}\left(1/2\right)$ (i.e., it admits values 0 and 1 with probability 1/2) and distribution of *X* is defined as $N\left(\mu Y,\Sigma \right)$, then *X* is a mixture of two normal distributions: $N\left(0,\Sigma \right)$ and $N\left(\mu ,\Sigma \right)$ with equal weights. Thus, in this section, we state auxiliary results on entropy of such random variable and its mutual information with its mixing distribution. The result for entropy of multivariate gaussian mixture, to the best of our knowledge, is new; for univariate case, it was derived in Reference [17]. Bounds and approximations of the entropy of a gaussian mixture are used, e.g., in signal processing; see, e.g., Reference [18,19]. Consider *d*-dimensional gaussian mixture *X* defined as

(20) $$X∼\frac{1}{2}N\left(0,I_{d}\right)+\frac{1}{2}N\left(\mu ,I_{d}\right),$$

where ‘∼’ signifies ‘distributed as’.

**Theorem** **1.** *Differential entropy of X in (20) equals*

$$H\left(X\right)=h\left(∥\mu ∥\right)+\frac{d-1}{2}\log\left(2\pi e\right),$$

*where h(a) is the differential entropy of one-dimensional gaussian mixture $2^{-1}\left\{N\left(0,1\right)+N\left(0,a\right)\right\}$ for a>0.*

(21) $$h\left(a\right)=-\int_{R}\frac{1}{2\sqrt{2\pi }}\left(e^{-\frac{x^{2}}{2}}+e^{-\frac{{\left(x-a\right)}^{2}}{2}}\right)\log\left(\frac{1}{2\sqrt{2\pi }}\left(e^{-\frac{x^{2}}{2}}+e^{-\frac{{\left(x-a\right)}^{2}}{2}}\right)\right)dx.$$

**Proof.**  In order to avoid burdensome notation, we prove the theorem for d=2 only. By the definition of differential entropy, we have

$$\begin{array}{cc} H(X) & =-\int_{R^{2}}\frac{1}{2}\left(f_{0}\left(x_{1},x_{2}\right)+f_{\mu }\left(x_{1},x_{2}\right)\right)\log\left(\frac{1}{2}\left(f_{0}\left(x_{1},x_{2}\right)+f_{\mu }\left(x_{1},x_{2}\right)\right)\right)dx_{1}dx_{2}, \end{array}$$

where *X* is defined in (20) for d=2, and $f_{\mu }$ denotes the density of normal distribution with a mean μ and a covariance matrix $I_{2}$.

We calculate the integral above changing the variables according to the following rotation

$$\begin{array}{c} \left(\begin{array}{c} y_{1}\\ y_{2} \end{array}\right)=\left(\begin{array}{cc} \frac{\mu_{1}}{∥\mu ∥} & -\frac{\mu_{2}}{∥\mu ∥}\\ \frac{\mu_{2}}{∥\mu ∥} & \frac{\mu_{1}}{∥\mu ∥} \end{array}\right)\left(\begin{array}{c} x_{1}\\ x_{2} \end{array}\right). \end{array}$$

Transformed densities $f_{0}$ and $f_{\mu }$ are equal

$$f_{0}\left(y_{1},y_{2}\right)=\frac{1}{2\pi }\exp\left(-\frac{y_{1}^{2}+y_{2}^{2}}{2}\right)$$

and

$$f_{\mu }\left(y_{1},y_{2}\right)=\frac{1}{2\pi }\exp\left(-\frac{{\left(y_{1}-∥\mu ∥\right)}^{2}+y_{2}^{2}}{2}\right).$$

Applying above transformation, we can decompose H(X) into sum of two integrals as follows:

$$\begin{array}{cc} H(X) & =\int_{R}\frac{1}{2\sqrt{2\pi }}\left(e^{-\frac{1}{2}y_{1}^{2}}+e^{-\frac{1}{2}{\left(y_{1}-∥\mu ∥\right)}^{2}}\right)\log\left(\frac{1}{2\sqrt{2\pi }}\left(e^{-\frac{1}{2}y_{1}^{2}}+e^{-\frac{1}{2}{\left(y_{1}-∥\mu ∥\right)}^{2}}\right)\right)dy_{1}\\ \; & +\int_{R}\frac{1}{\sqrt{2\pi }}e^{-\frac{1}{2}y_{2}^{2}}\log\left(\frac{1}{\sqrt{2\pi }}e^{-\frac{1}{2}y_{2}^{2}}\right)dy_{2}=h\left(∥\mu ∥\right)+\frac{1}{2}\log\left(2\pi e\right), \end{array}$$

where in the last equality the value H(Z)=log(2πe)/2 for N(0,1) variable *Z* is used. This ends the proof. □

The result above is now generalized to the case of arbitrary covariance matrix Σ. The general case will follow from Theorem 1 and the scaling property of differential entropy under linear transformations.

**Theorem** **2.** *Differential entropy of*

$$X∼\frac{1}{2}N\left(0,\Sigma \right)+\frac{1}{2}N\left(\mu ,\Sigma \right)$$

*equals*

$$H\left(X\right)=h\left(∥\Sigma^{-1/2}\mu ∥\right)+\frac{d-1}{2}\log\left(2\pi e\right)+\frac{1}{2}\log\left(\det\Sigma \right).$$

**Proof.**  We apply Theorem 1 to multivariate random variable $Y=\Sigma^{-\frac{1}{2}}X$. We obtain

$$\begin{array}{c} H\left(Y\right)=h\left(∥\Sigma^{-1/2}\mu ∥\right)+\frac{d-1}{2}\log\left(2\pi e\right). \end{array}$$

Using the scaling property of differential entropy [6], we have

$$\begin{array}{c} H\left(X\right)=H\left(Y\right)+\frac{1}{2}\log\left(\det\Sigma \right), \end{array}$$

which completes the proof. □

Similarly, we obtain the formula for mutual information of gaussian mixture and its mixing distribution. We use shorthand X|Y=y to denote random variable defined as having distribution coinciding with conditional distribution P(X|Y=y).

**Theorem** **3.** *Mutual information of X and Y where $Y∼\mathrm{Bern}\left(1/2\right)$ and $X|Y=y∼N\left(y\mu ,\Sigma \right)$ equals*

(22) $$I\left(X,Y\right)=h\left(∥\Sigma^{-1/2}\mu ∥\right)-\frac{1}{2}\log\left(2\pi e\right).$$

**Proof.**  We will use here the fact that the entropy of multidimensional normal distribution $Z∼N\left(\mu_{Z},\Sigma \right)$ equals (cf. Reference [6], Theorem 8.4.1)

$$H\left(Z\right)=\frac{d}{2}\log\left(2\pi e\right)+\frac{1}{2}\log\left(\det\Sigma \right).$$

Therefore, we have

(23) $$I\left(X,Y\right)=H\left(X\right)-H\left(X|Y\right)=h\left(∥\Sigma^{-1/2}\mu ∥\right)-\frac{1}{2}\log\left(2\pi e\right),$$

as

(24) $$H\left(X|Y\right)=\frac{1}{2}H\left(X|Y=0\right)+\frac{1}{2}H\left(X|Y=1\right),$$

where H(X|Y=i) stands for the entropy of *X* on the stratum Y=i. We notice that H(X|Y=i)=H(Z), as the distribution of *X* on stratum Y=i is normal with covariance matrix Σ, and its entropy does not depend on the mean. □

**Figure 1:**
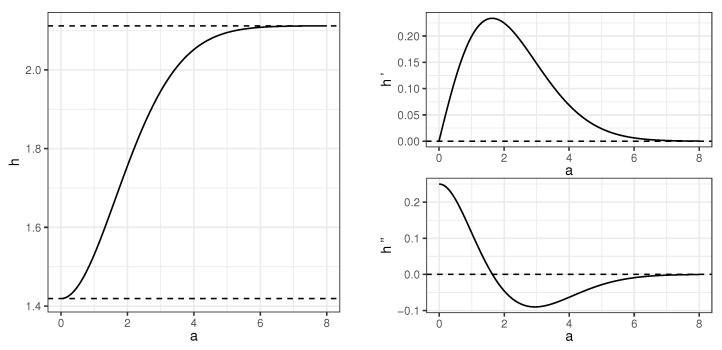
Behavior of function *h* and its two first derivatives. Horizontal lines in the left chart correspond to bounds of *h* and equal $\frac{1}{2}\log\left(2\pi e\right)$ and $\frac{1}{2}\log\left(2\pi e\right)+\log\left(2\right)$, respectively.

We note that, in Reference [17], entropy of one-dimensional Gaussian mixture $2^{-1}\left(N\left(a,1\right)+N\left(-a,1\right)\right)$ is calculated as $h_{e}\left(a\right)$, where $h_{e}\left(a\right)$ is given in an integral form. As the entropy is invariant with respect to translation, function h(a) defined above equals $h_{e}\left(a/2\right)$. The behavior of *h* and its two first derivatives is shown in Figure 1. It indicates that the function *h* is strictly increasing, and this fact is also stated in Reference [17] without proof. This is proved formally below. Strict monotonicity of *h* plays a crucial role in determining the order in which variables are included in a set of active variables. Note that h(0)=log(2πe)/2, which is the entropy of the standard normal N(0,1) variable. Values of *h* need to be calculated numerically.

**Lemma** **1.** *Differential entropy h(a) of gaussian mixture defined in Theorem 1 is strictly increasing function of a.*

**Proof.**  It is easy to see that *h* is differentiable and for calculation of its derivative, integration in (21) and taking derivatives can be interchanged. We show that derivative of *h* is positive. We have by standard manipulations, using the fact that $x\exp(-x^{2}/2)$ is an odd function for the second equality below, that

$$\begin{array}{cc} h^{\prime }\left(a\right) & =-\frac{1}{2\sqrt{2\pi }}\int_{R}\left(\left(x-a\right)e^{-\frac{{\left(x-a\right)}^{2}}{2}}\log\left(\frac{1}{2\sqrt{2\pi }}\left(e^{-\frac{x^{2}}{2}}+e^{-\frac{{\left(x-a\right)}^{2}}{2}}\right)\right)+\left(x-a\right)e^{-\frac{{\left(x-a\right)}^{2}}{2}}\right)dx\\ \; & =-\frac{1}{2\sqrt{2\pi }}\int_{R}\left(x-a\right)e^{-\frac{{\left(x-a\right)}^{2}}{2}}\log\left(\frac{1}{2\sqrt{2\pi }}\left(e^{-\frac{x^{2}}{2}}+e^{-\frac{{\left(x-a\right)}^{2}}{2}}\right)\right)dx\\ \; & =-\frac{1}{2\sqrt{2\pi }}\int_{R}xe^{-\frac{x^{2}}{2}}\log\left(\frac{1}{2\sqrt{2\pi }}\left(e^{-\frac{x^{2}}{2}}+e^{-\frac{{\left(x+a\right)}^{2}}{2}}\right)\right)dx\\ \; & =-\frac{1}{2\sqrt{2\pi }}\int_{0}^{\infty }xe^{-\frac{x^{2}}{2}}\log\left(\frac{1}{2\sqrt{2\pi }}\left(e^{-\frac{x^{2}}{2}}+e^{-\frac{{\left(x+a\right)}^{2}}{2}}\right)\right)dx\\ \; & \;-\frac{1}{2\sqrt{2\pi }}\int_{-\infty }^{0}xe^{-\frac{x^{2}}{2}}\log\left(\frac{1}{2\sqrt{2\pi }}\left(e^{-\frac{x^{2}}{2}}+e^{-\frac{{\left(x+a\right)}^{2}}{2}}\right)\right)dx\\ \; & =\frac{1}{2\sqrt{2\pi }}\int_{0}^{\infty }xe^{-\frac{x^{2}}{2}}\left(\log\left(\frac{1}{2\sqrt{2\pi }}\left(e^{-\frac{x^{2}}{2}}+e^{-\frac{{\left(x-a\right)}^{2}}{2}}\right)\right)-\log\left(\frac{1}{2\sqrt{2\pi }}\left(e^{-\frac{x^{2}}{2}}+e^{-\frac{{\left(x+a\right)}^{2}}{2}}\right)\right)\right)dx. \end{array}$$

We have used change of variables for the third and the fifth equality above. It follows from the last expression that $h^{\prime }\left(a\right)>0$ as ${\left(x-a\right)}^{2}<{\left(x+a\right)}^{2}$ for x>0 and a>0, and, therefore, *h* is increasing. □

**Remark** **1.** *Note that Theorems 2 and 3 in conjunction with Lemma 1 show that entropy of mixture of two gaussians with the same covariance matrix and its mutual information with mixing distribution is strictly increasing function of the norm $∥\Sigma^{-1}\mu ∥$. In particular, for Σ=I, entropy increases as the distance between centers of two gaussians increases. In addition, it follows from (22) and I(X,Y)≥0 that h(s)≥log(2πe)/2 for any s∈R.*

**Remark** **2.** *We call a random variable $X\in R^{d}$ a generalized mixture when there exist diffeomorphisms $f_{i}:R\to R$ such that $\left(f_{1}\left(X_{1}\right),\ldots f_{p}\left(X_{d}\right)\right)∼2^{-1}\left(N\left(0,I_{d}\right)+N\left(\mu ,I_{d}\right)\right)$. Then, it follows from Theorem 2 that, analogously to Reference [20], that total correlation of X (cf. Reference [21]) defined as $T\left(X\right)=\sum_{i=1}^{d}H\left(X_{i}\right)-H\left(X\right)$ equals for generalized mixture X*

$$TC\left(X\right)=\sum_{i=1}^{d}h(|\mu_{i}\left|\right)-h\left(||\mu ||\right)+\left(1-d\right)\log\left(2\pi e\right)/2,$$

*where $\mu ={\left(\mu_{1},\ldots ,\mu_{d}\right)}^{T}$.*

## 4. Main Results: Behavior of Information-Based Criteria in Generative Tree Model

In the following, we define a special gaussian Generative Tree Model and investigate how greedy procedure based on (14), as well as its analogues when CMI is replaced by JMI and CIFE, behaves in this model. Theorem 22 proved in the previous section will yield explicit formulae for CMIs in this model, whereas strict monotonicity of function h(·) proved in Lemma 1 will be essential to compare values of $I(X_{j},Y|X_{S})$ for different candidates $X_{j}$.

### 4.1. Generative Tree Model

**Figure 2:**
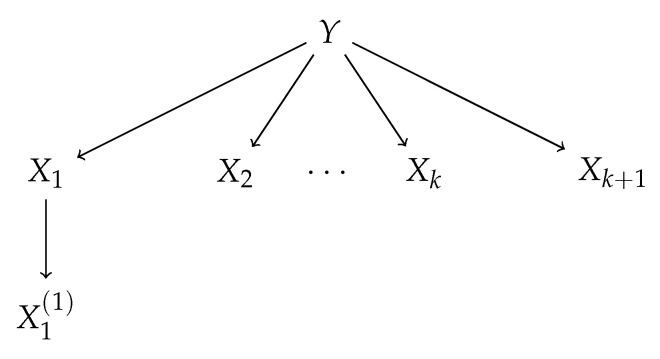
Generative Tree Model under consideration.

We will consider the Generative Tree Model with tree structure illustrated in the Figure 2. Data Generating Process described by this model yields the distribution of the random vector $\left(Y,X_{1},\ldots ,X_{k+1},X_{1}^{\left(1\right)}\right)$ such that:

(25) $$\begin{array}{c} Y∼\mathrm{Bern}\left(1/2\right),\;X_{i}\left|Y∼N\left(\gamma^{i-1}Y,1\right)\mathrm{and}i\in \left\{1,2,\ldots ,k+1\right\},\;\right|X_{1}∼N\left(X_{1},1\right), \end{array}$$

where 0<γ≤1 is the parameter. Thus, first the value Y=0,1 is generated with both values 0 and 1 having the same probability 1/2; then, $X_{1},\ldots X_{k+1}$ are generated as normal variables with the variance 1 and the mean equal to *Y*. Finally, once the value of $X_{1}$ is obtained, $X_{1}^{\left(1\right)}$ is generated from normal distribution with the variance 1 and the mean equal to $X_{1}$. Thus, in the sense specified above, $X_{1},\ldots X_{k+1}$ are the children of *Y* and $X_{1}^{\left(1\right)}$ is the child of $X_{1}$. Parameter γ controls how difficult the problem of feature selection is. Namely, the smaller the parameter γ is, the less information $X_{i}$ holds about *Y* for i∈{1,2,…,k+1}. We will refer to the model defined above as $M_{k,\gamma }$. We denote by, abusing slightly the notation, $p\left(y,x_{i}\right),p\left(x_{1},x_{1}^{\left(1\right)}\right)$ bivariate densities and by $p\left(y\right),p\left(x_{i}\right),p\left(x_{1}^{\left(1\right)}\right)$ marginal densities. With this notation, the joint density $p(y,x_{1},\ldots ,x_{k+1},x_{1}^{\left(1\right)})$ equals

$$p\left(y\right)\left[\prod_{i=1}^{k+1}\frac{p(y,x_{i})}{p(y)}\right]\frac{p(x_{1},x_{1}^{\left(1\right)})}{p(x_{1})}=\frac{p(x_{1},x_{1}^{\left(1\right)})}{p\left(x_{1}\right)p\left(x_{1}^{\left(1\right)}\right)}\prod_{i=1}^{k+1}\frac{p(y,x_{i})}{p\left(y\right)p\left(x_{i}\right)}\left[\prod_{i=1}^{k+1}p\left(x_{i}\right)\right]p\left(y\right)p\left(x_{1}^{\left(1\right)}\right),$$

which can be more succinctly written as

$$\prod_{(i,j)\in E}\frac{p(z_{i},z_{j})}{p\left(z_{i}\right)p\left(z_{j}\right)}\prod_{i\in V}p\left(z_{i}\right),$$

after renaming the variables to $z_{i},\;i=1,\ldots k+3$ and *E* and *V* standing for edges and vertices in the graph shown in Figure 2 (cf. formula (4.1) in Reference [4]).

**Figure 3:**
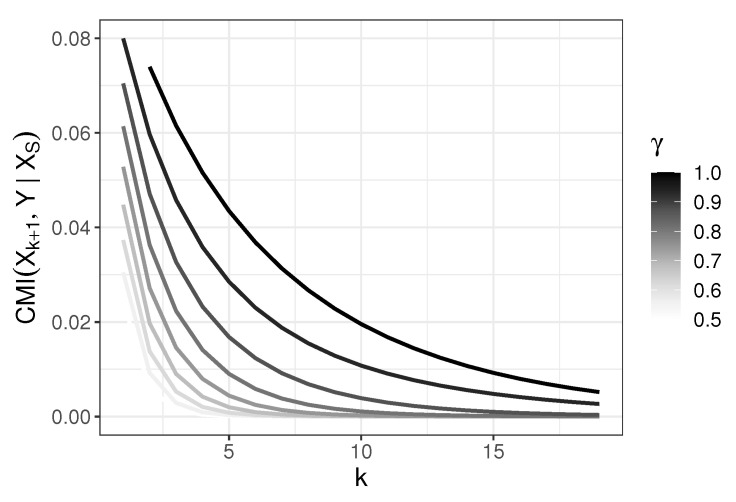
Behavior of conditional mutual information $I(X_{k+1},Y|X_{1},X_{2},\ldots ,X_{k})$ as a function of *k* for different γ values.

The above model generalizes the model discussed in Reference [3], but some branches which are irrelevant in our considerations are omitted. The values of conditional mutual information $I(X_{k+1},Y|X_{S})$ in the model, where S={1,2,…,k} for different γ as a function of *k* are shown in the Figure 3. We prove in the following that $I(X_{k+1},Y|X_{S})>0$; thus, $X_{k+1}$ carries non-null predictive information about *Y* even when variables $X_{1},\ldots ,X_{k}$ are already chosen as predictors. We note that $I(X_{1}^{\left(1\right)},Y|X_{S})=0$ for every γ∈(0,1] and $X_{S}$ containing $X_{1}$. Thus, $\left\{X_{1},\ldots ,X_{k+1}\right\}$ is the Markov Blanket (cf., e.g., Reference [22]) of *Y* among predictors $\left\{X_{1},\ldots ,X_{k+1},X_{1}^{\left(1\right)}\right\}$ and $\left\{X_{1},\ldots ,X_{k+1}\right\}$ is sufficient for *Y* (cf. Reference [23]). A more general model may be considered which incorporates children of every vertex $X_{1},\ldots ,X_{k+1}$, and several levels of progeny. Here, we show how one variable $X_{1}^{\left(1\right)}$ which does not belong to Markov Blanket of *Y* is treated differently by the considered selection rules.

Intuitively, for 0<γ<1 and l<n$X_{l}$ carry more information about *Y* than $X_{n}$ and, moreover, $X_{1}^{\left(1\right)}$ is redundant once $X_{1}$ has been chosen. Thus, predictors should be chosen in order $X_{1},X_{2},\ldots X_{k+1}$. For γ=1, the order of selection of $X_{i}$ is also $X_{1},\ldots ,X_{k+1}$ in concordance with our convention of breaking ties, but $X_{1}^{\left(1\right)}$ should not be chosen. We show in the following that CMI chooses variables in this order; however, the order with respect to its approximations, CIFE, and JMI may be different. We also note that alternative way of representing predictors is

(26) $$X_{i}=\gamma^{i-1}Y+\varepsilon_{i},\;\;X_{1}^{\left(1\right)}=X_{1}+\varepsilon_{k+2},$$

for i=1,…,k+1, where $\varepsilon_{1},\ldots ,\varepsilon_{k+2}$ are i.i.d. N(0,1). Thus, in particular

$$a_{k}Y=\sum_{i=1}^{k+1}X_{i}-\sum_{i=1}^{k+1}\varepsilon_{i},$$

with $a_{k}=\left(1-\gamma^{k+1}\right)/\left(1-\gamma \right)$. Moreover, it is seen that $EX_{i}=\gamma^{i-1}EY=\gamma^{i-1}/2$.

It is shown in Reference [2] that maximization of $I(X_{j},Y|X_{S})$ is equivalent to maximization of $CIFE(X_{j},Y|X_{S})$ provided that selected features in $X_{S}$ are independent and class-conditionally independent given unselected features $X_{j}$. It is easily seen that these properties do not hold in the considered GTM for S={1,…,l} and j=l+1 for l≤k. It can also be seen by a direct calculation that CMI differs from CIFE in GTM. Take S={1,2} and $X_{j}=X_{1}^{\left(1\right)}$. Then, note that the difference between these quantities equals

(27) $$I\left(X_{j},Y|X_{S}\right)-I\left(X_{j},Y\right)-\sum_{i\in S}II\left(X_{i},X_{j},Y\right)$$

Moreover, using conditional independence, we have

$$II\left(X_{1},X_{1}^{\left(1\right)},Y\right)=I\left(X_{1}^{\left(1\right)},Y|X_{1}\right)-I\left(X_{1}^{\left(1\right)},Y\right)=-I\left(X_{1}^{\left(1\right)},Y\right)$$

and

$$II\left(X_{2},X_{1}^{\left(1\right)},Y\right)=I\left(X_{1}^{\left(1\right)},X_{2}|Y\right)-I\left(X_{1}^{\left(1\right)},X_{2}\right)=-I\left(X_{1}^{\left(1\right)},X_{2}\right);$$

thus, plugging the above equalities into (27) and using $I(X_{1}^{\left(1\right)},Y|X_{1},X_{2})=0$, we obtain that expression there equals $I(X_{1}^{\left(1\right)},X_{2})$, which is strictly positive in the considered GTM.

Similar considerations concerning conditions stated above (18) show that maximization of JMI is not equivalent to maximization of CMI in GTM. Namely, if S={1,2} and j∈{3,…,k+1}, then it is easily seen that $I(X_{j},X_{S\backslash \{i\}}|X_{i})>0$ and $I(X_{j},X_{S\backslash \{i\}}|X_{i},Y)=0$ for i=1,2; thus, the last term in (17) is negative.

In order to support this numerically for a specific case, consider γ=2/3. In the first column of the Table 1a, MI values $I(X_{i},Y),i=1,\ldots ,4$ are shown for this value of γ. They were calculated in Reference [3] using simulations, while here they are based on (23) and numerical evaluation of $h\left(∥\Sigma^{-1/2}\mu ∥\right)$. Additionally, in Table 1, CMI values from subsequent steps and JMI and CIFE values in such a model are shown. As a foretaste of the analysis which follows, note that, in view of panel (b) of the table, JMI chooses erroneously $X_{1}^{\left(1\right)}$ in the third step instead of $X_{3}$ in contrast to CIFE (cf. part (c) of the table) which chooses $X_{1},X_{2},X_{3}$ in the right order. Note also that, in this case, is the second largest mutual informations with *Y*; thus, when the filter based solely on this information is considered, then $X_{1}^{\left(1\right)}$ is chosen at the second step (after $X_{1}$).

We note that analysis of behavior of CMI and its approximations including CIFE and JMI has been given in Reference [24], Section 6, for a simple model containing 4 predictors. We analyze here the behavior of these measures of conditional dependence for the general model $M_{k,\gamma }$, which involves arbitrary number of predictors having varying dependence with *Y*.

### 4.2. Behavior of CMI

First of all, we show that the criterion based on conditional mutual information CMI without any modifications chooses correct variables in the right order. It has been previously noticed that $I(X_{1}^{\left(1\right)},Y|X_{S})=0$ for S={1,…,k}. Now, we show that $I(X_{k+1},Y|X_{S})>0$ for every *k*. Namely, applying Theorem 3 and the chain rule for mutual information

$$I\left(X_{S\cup \{k+1\}},Y\right)=I\left(X_{S},Y\right)+I\left(X_{k+1},Y|X_{S}\right),$$

we obtain

(28) $$I\left(X_{k+1},Y|X_{S}\right)=h\left(\sqrt{\sum_{i=0}^{k}\gamma^{2i}}\right)-h\left(\sqrt{\sum_{i=0}^{k-1}\gamma^{2i}}\right)>0,$$

where the inequality follows as *h* is an strictly increasing function. Thus, we proved that $I\left(X_{1}^{\left(1\right)},Y|X_{S}\right)=0<I\left(X_{k+1},Y|X_{S}\right)$ for S={1,…,k} for every *k*. Whence we have for S={1,…,l} and l<k that

$$\underset{Z\in S^{c}}{arg max}I\left(Z,Y|X_{S}\right)=X_{l+1},$$

thus CMI chooses predictors in a correct order. Figure 3 shows behavior of $g\left(k,\gamma \right)=I\left(X_{k+1},Y|X_{1},\ldots ,X_{k}\right)$ as the function of *k* for various γ. Note that it follows from Figure 3 that g(·,γ) is decreasing. This means that the additional information on *Y* obtained when $X_{k+1}$ is incorporated gets smaller with *k*. Now, we study the order in which predictors are chosen with respect to JMI and CIFE.

### 4.3. Behavior of JMI

The main objective of this section is to examine performance of JMI criterion in the Generative Tree Model for different values of parameter γ. We will show that:

- For γ=1 active predictors $X_{1},\ldots ,X_{k+1}\in MB\left(Y\right)$ are chosen in the right order and $X_{1}^{\left(1\right)}$ is not chosen before them;
- For 0<γ<1, variable $X_{1}^{\left(1\right)}\notin MB\left(Y\right)$ is chosen at a certain step before all $X_{1},\ldots ,X_{k+1}$ are chosen, and we evaluate a moment when this situation occurs.

Consider the model above and assume that the set of indices of currently chosen variables equals S={1,2,…,k}. For i∈{1,2,…,k} we apply chain rule (6) and Theorem 3 with the following covariance matrices and mean vectors for $I(\left(X_{i},Z\right),Y)$ (cf. (26)):

(29) $$\Sigma =\left(\begin{array}{cc} 1 & 0\\ 0 & 1 \end{array}\right),\mu =\left(\begin{array}{c} \gamma^{i-1}\\ \gamma^{k} \end{array}\right)\mathrm{and}\Sigma =\left(\begin{array}{cc} 1 & 0\\ 0 & 2 \end{array}\right),\mu =\left(\begin{array}{c} \gamma^{i-1}\\ 1 \end{array}\right),$$

respectively, for $Z=X_{k+1}$ and $Z=X_{1}^{\left(1\right)}$. Then, we have

(30) $$\begin{array}{cc} I(X_{k+1},Y|X_{i}) & =h\left(\sqrt{\gamma^{2k}+\gamma^{2(i-1)}}\right)-h\left(\gamma^{i-1}\right), \end{array}$$

(31) $$\begin{array}{cc} I(X_{1}^{\left(1\right)},Y|X_{i}) & =h\left(\sqrt{\gamma^{2(i-1)}+\frac{1}{2}}\right)-h\left(\gamma^{i-1}\right)\mathrm{for}i\neq 1, \end{array}$$

(32) $$\begin{array}{cc} I(X_{1}^{\left(1\right)},Y|X_{1}) & =0. \end{array}$$

The last equation follows from the fact that $X_{1}^{\left(1\right)}$ and *Y* are conditionally independent given $X_{1}$.

From the definition of $JMI(X,Y|X_{S})$, abbreviated from now on to $JMI(X|X_{S})$ to simplify notation, we obtain

(33) $$\begin{array}{cc} kJMI(X_{k+1}|X_{S}) & =\sum_{i=1}^{k}\left(h\left(\sqrt{\gamma^{2k}+\gamma^{2(i-1)}}\right)-h\left(\gamma^{i-1}\right)\right), \end{array}$$

(34) $$\begin{array}{cc} kJMI(X_{1}^{\left(1\right)}|X_{S}) & =\left\{\begin{array}{cc} 0 & \mathrm{if}\;k=1\\ \sum_{i=2}^{k}\left(h\left(\sqrt{\gamma^{2(i-1)}+\frac{1}{2}}\right)-h\left(\gamma^{i-1}\right)\right) & \mathrm{if}\;k>1 \end{array}\right.. \end{array}$$

We observe that the variables $X_{1},X_{2},\ldots$ are chosen in order according to JMI, as for S={1,…,l} and l<m<n, we have $JMI\left(X_{m}\right)>JMI\left(X_{n}\right)$. For γ=1, the right-hand sides of the last two expressions equal $k\left(h\left(\sqrt{2}\right)-h\left(1\right)\right)$ and $\left(k-1\right)\left(h\left(\sqrt{3/2}\right)-h\left(1\right)\right)$, respectively. Thus, for γ=1, we have $JMI\left(X_{k+1}|X_{S}\right)>JMI\left(X_{1}^{\left(1\right)}|X_{S}\right)$, which means that variables are chosen in the order $X_{1},\ldots ,X_{k+1}$ and $X_{1}^{\left(1\right)}$ is not chosen before them when JMI criterion is used. Although, for γ=1, JMI criterion does not select this redundant feature, we note that, for k→∞, S={1,…,k}, and γ=1

$$JMI\left(X_{1}^{\left(1\right)}|X_{S}\right)\to \left(h\left(\sqrt{\frac{3}{2}}\right)-h\left(1\right)\right)>0,$$

which differs from $I(X_{1}^{\left(1\right)},Y|X_{S})=0$ for all k≥1. We note also that, in this case, $JMI(X_{k+1}|X_{S})$ does not depend on *k* in contrast to $I(X_{k+1},Y|X_{S})$.

Now, we will consider the case 0<γ<1. We want to show that, for sufficiently large *k* and S={1,…,k}, JMI criterion chooses $X_{1}^{\left(1\right)}$ since

$$JMI\left(X_{k+1}|X_{S}\right)<JMI\left(X_{1}^{\left(1\right)}|X_{S}\right).$$

The last inequality is equivalent to

(35) $$\sum_{i=2}^{k}\left(h\left(\sqrt{\gamma^{2(i-1)}+\frac{1}{2}}\right)-h\left(\sqrt{\gamma^{2k}+\gamma^{2(i-1)}}\right)\right)>h\left(\sqrt{1+\gamma^{2k}}\right)-h\left(1\right).$$

The right-hand side tends to 0 when k→∞. For the left-hand side, note that, for $k>-\frac{\log_{\gamma }2}{2}$, we have $\gamma^{2k}<1/2$, and all summands of the sum above are positive, as *h* is an increasing function. Thus, bounding the sum by its first term, we have

$$\begin{array}{c} \sum_{i=2}^{k}\left(h\left(\sqrt{\gamma^{2(i-1)}+\frac{1}{2}}\right)-h\left(\sqrt{\gamma^{2k}+\gamma^{2(i-1)}}\right)\right)>h\left(\sqrt{\gamma^{2}+1/2}\right)-h\left(\sqrt{\gamma^{2}+1/2}\right)=0. \end{array}$$

**Figure 4:**
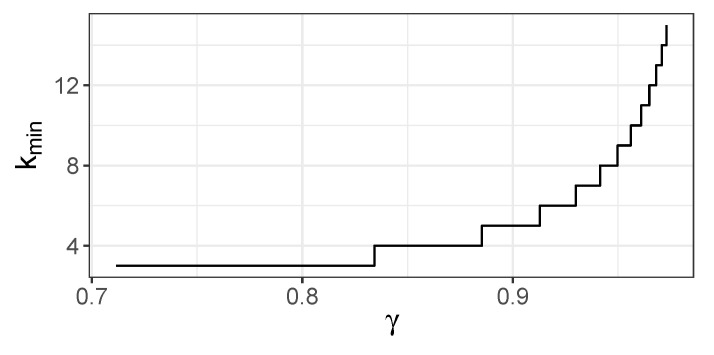
Minimal *k* for which $JMI\left(X_{k+1}|X_{S}\right)<JMI\left(X_{1}^{\left(1\right)}|X_{S}\right)$, 0<γ<1.

**Figure 5:**
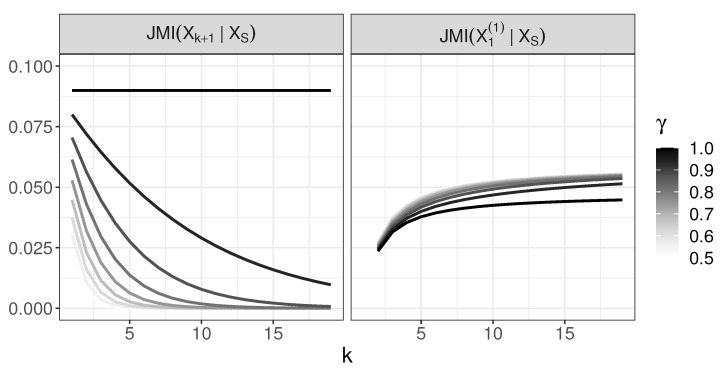
The behavior of JMI in the generative tree model: $JMI(X_{k+1}|X_{S})$ and $JMI(X_{1}^{\left(1\right)}|X_{S})$.

The minimal *k* for which the JMI criterion incorrectly chooses $X_{1}^{\left(1\right)}$, i.e., the first *k* for which (35) holds, is shown in Figure 4. The values of JMI criterion for variables $X_{k+1}$ and $X_{1}^{\left(1\right)}$ is shown in Figure 5. Figure 4 indicates that $X_{1}^{\left(1\right)}$ is chosen early; for γ≤0.8, it happens in the third step at the latest.

### 4.4. Behavior of CIFE and Its Comparison with JMI

The aim of this section is to show that, although both JMI and CIFE criteria are developed as approximations to conditional mutual information, their behavior in the tree generative model differs. We will show that:

- For γ=1, CIFE incorrectly chooses $X_{1}^{\left(1\right)}$ at some point;
- For 0<γ<1, CIFE selects variables $X_{1},\ldots ,X_{k+1}$ in the right order.

Thus, CIFE behaves very differently from JMI in Generative Tree Model.

Analogously to formulae for JMI, we have the following formulae for CIFE (S={1,…,k}):

$$\begin{array}{cc} CIFE(X_{k+1}|X_{S}) & =\left(1-k\right)\left(h\left(\gamma^{k}\right)-\frac{1}{2}\log\left(2\pi e\right)\right)+\sum_{i=1}^{k}\left(h\left(\sqrt{\gamma^{2k}+\gamma^{2(i-1)}}\right)-h\left(\gamma^{i-1}\right)\right),\\ CIFE(X_{1}^{\left(1\right)}|X_{S}) & =\left\{\begin{array}{cc} 0 & \mathrm{if}\;k=1\\ \left(1-k\right)\left(h\left(1\right)-\frac{1}{2}\log\left(2\pi e\right)\right)+\sum_{i=2}^{k}\left(h\left(\sqrt{\gamma^{2(i-1)}+\frac{1}{2}}\right)-h\left(\gamma^{i-1}\right)\right) & \mathrm{if}\;k>1 \end{array}\right.. \end{array}$$

For γ=1, we have

$$\begin{array}{cc} CIFE(X_{k+1}|X_{S}) & =\left(1-k\right)\left(h\left(1\right)-\frac{1}{2}\log\left(2\pi e\right)\right)+\sum_{i=1}^{k}\left(h\left(\sqrt{2}\right)-h\left(1\right)\right),\\ \; & =h\left(1\right)-\frac{1}{2}\log\left(2\pi e\right)-k\left(2h\left(1\right)-h\left(\sqrt{2}\right)-\frac{1}{2}\log\left(2\pi e\right)\right)\\ CIFE(X_{1}^{\left(1\right)}|X_{S}) & =\left(1-k\right)\left(2h\left(1\right)-\frac{1}{2}\log\left(2\pi e\right)-h\left(\sqrt{\frac{3}{2}}\right)\right). \end{array}$$

**Figure 6:**
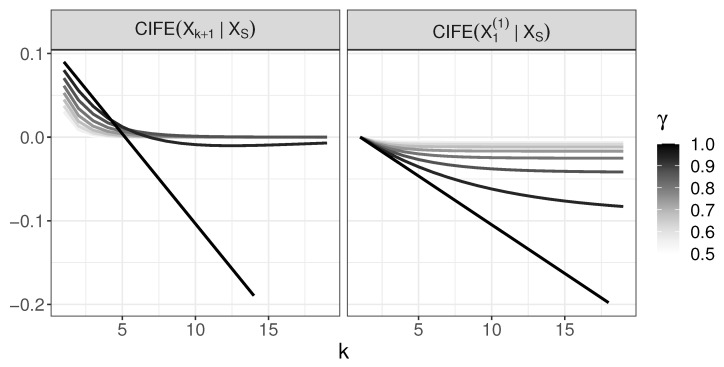
The behavior of CIFE in the generative tree model: $CIFE(X_{k+1}|X_{S})$ and $CIFE(X_{1}^{\left(1\right)}|X_{S})$.

**Figure 7:**
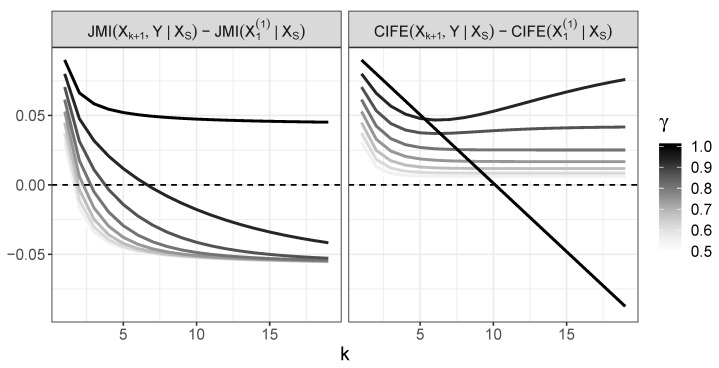
Difference between values of JMI for $X_{k+1}$ and $X_{1}^{\left(1\right)}$ (**left panel**) and analogous difference for CIFE (**right panel**). Values below 0 mean that the variable $X_{1}^{\left(1\right)}$ is chosen.

Note that both expressions above are linear functions with respect to *k*. Comparison of their slopes, in view of $h\left(\sqrt{\frac{3}{2}}\right)<h\left(\sqrt{2}\right)$ as *h* is an increasing function, yields that, for sufficiently large *k*, we obtain $CIFE\left(X_{k+1}|X_{S}\right)<CIFE\left(X_{1}^{\left(1\right)}|X_{S}\right)$. The behavior of CIFE for 0<γ<1 in case of $X_{k+1}$ and $X_{1}^{\left(1\right)}$ is shown in Figure 6 and the difference between $CIFE(X_{k+1}|X_{S})$ and $CIFE(X_{1}^{\left(1\right)}|X_{S})$ in Figure 7. The values below 0 in the last plot occur for γ=1; only, thus, for 0<γ<1, we have $CIFE\left(X_{k+1}|X_{S}\right)>CIFE\left(X_{1}^{\left(1\right)}|X_{S}\right)$ for any *k*.

Furthermore, as $2h\left(1\right)-\frac{1}{2}\log\left(2\pi e\right)-h\left(\sqrt{\frac{3}{2}}\right)\approx 0.0642>0$, we have, for γ=1,

$$CIFE(X_{1}^{\left(1\right)}|X_{S})\to -\infty \mathrm{as}k\to \infty ,$$

and as $2h\left(1\right)-h\left(\sqrt{2}\right)-\frac{1}{2}\log\left(2\pi e\right)\approx 0.0215>0$, we have

$$CIFE(X_{k+1}|X_{S})\to -\infty \mathrm{as}k\to \infty .$$

In order to understand the consequences of this property, let us momentarily assume that one introduces an intuitive stopping rule which says that candidate $X_{j_{0}}$ such that $j_{0}={arg max}_{j\in S^{c}}CIFE\left(X_{j},Y|X_{S}\right)$ is appended only when $CIFE(X_{j_{0}},Y|X_{S})>0$. Then, Positive Selection Rate (PSR) of such selection procedure may become arbitrarily small in model $M_{k,\gamma }$ for fixed γ and sufficiently large *k*. PSR is defined as $|\hat{t}\cap t|/|t|$, where t={1,…,k+1} is a set of indices of Markov Blanket of *Y* and $\hat{t}$ is a set of indices of the chosen variables.

## 5. Conclusions

We have considered $M_{k,\gamma }$, a special case of Generative Tree Model and investigated behavior of CMI and related criteria JMI and CIFE in this model. We have shown that, despite the fact that both of these criteria are derived as approximations of CMI under certain dependence conditions, their behavior may greatly differ from that of CMI in the sense that they may switch the order of variable importance and treat inactive variables as more relevant than active ones. In particular, this occurs for JMI when γ<1 and CIFE for γ=1. We have also shown a drawback of CIFE procedure which consists in disregarding significant part of active variables so that PSR may become arbitrarily small in model $M_{k,\gamma }$ for large *k*. As a byproduct, we obtain formulae for the entropy of multivariate gaussian mixture and its mutual information with mixing variable. We have also shown that the entropy of the gaussian mixture is a strictly increasing function of the euclidean distance between two centers of its components. Note that, in this paper, we investigated behavior of theoretical CMI and its approximations in GTM; for their empirical versions, we may expect exacerbation of effects described here.

## Figures and Tables

**Table 1:** The criteria (Conditional Mutual Information (CMI), Joint Mutual Information (JMI), Conditional Infomax Feature Extraction (CIFE)) values for k=2 and γ=2/3. A value of the chosen variable in each step and for each criterion is in bold.

(**a**) $X_{S_{1}}=\left\{X_{1}\right\}$, $X_{S_{2}}=\left\{X_{1},X_{2}\right\}$, $X_{S_{3}}=\left\{X_{1},X_{2},X_{3}\right\}$				
	I(·,Y)	$I(\cdot ,Y|X_{S_{1}})$	$I(\cdot ,Y|X_{S_{2}})$	$I(\cdot ,Y|X_{S_{3}})$
$X_{1}$	**0.1114**			
$X_{2}$	0.0527	**0.0422**		
$X_{3}$	0.0241	0.0192	**0.0176**	
$X_{1}^{\left(1\right)}$	0.0589	0.0000	0.0000	**0.0000**
(**b**) $X_{S_{1}}=\left\{X_{1}\right\}$, $X_{S_{2}}=\left\{X_{1},X_{2}\right\}$, $X_{S_{3}}=\left\{X_{1},X_{2},X_{1}^{\left(1\right)}\right\}$				
	JMI(·)	$JMI(\cdot |X_{S_{1}})$	$JMI(\cdot |X_{S_{2}})$	$JMI(\cdot |X_{S_{3}})$
$X_{1}$	**0.1114**			
$X_{2}$	0.0527	**0.0422**		
$X_{3}$	0.0241	0.0192	0.0205	**0.0208**
$X_{1}^{\left(1\right)}$	0.0589	0.0000	**0.0266**	
(**c**) $X_{S_{1}}=\left\{X_{1}\right\}$, $X_{S_{2}}=\left\{X_{1},X_{2}\right\}$, $X_{S_{3}}=\left\{X_{1},X_{2},X_{3}\right\}$				
	CIFE(·)	$CIFE(\cdot |X_{S_{1}})$	$CIFE(\cdot |X_{S_{2}})$	$CIFE(\cdot |X_{S_{3}})$
$X_{1}$	**0.1114**			
$X_{2}$	0.0527	**0.0422**		
$X_{3}$	0.0241	0.0192	**0.0169**	
$X_{1}^{\left(1\right)}$	0.0589	0.0000	−0.0057	**−0.0083**
